# Imaging cardiac innervation in hereditary transthyretin (ATTRm) amyloidosis: A marker for neuropathy or cardiomyopathy in case of heart failure?

**DOI:** 10.1007/s12350-018-01477-y

**Published:** 2018-10-29

**Authors:** Daphne L. Jonker, Bouke P. C. Hazenberg, Hans L. A. Nienhuis, Riemer H. J. A. Slart, Andor W. J. M. Glaudemans, Walter Noordzij

**Affiliations:** 1 Department of Nuclear Medicine and Molecular Imaging, Medical Imaging Center, University of Groningen, University Medical Center Groningen, Groningen, The Netherlands; 2 Department of Rheumatology and Clinical Immunology, University of Groningen, University Medical Center Groningen, Groningen, The Netherlands; 3 Department of Biomedical Photonic Imaging, University of Twente, Enschede, The Netherlands

**Keywords:** Cardiac amyloidosis, ATTRm, autonomic function tests, cardiac biomarkers, bone scintigraphy, MIBG

## Abstract

**Background:**

Nuclear imaging modalities using ^123^Iodine-metaiodobenzylguanidine (^123^I-MIBG) and bone seeking tracers identify early cardiac involvement in ATTRm amyloidosis patients. However, little is known whether results from ^123^I-MIBG scintigraphy actually correlate to markers for either cardiac autonomic neuropathy or cardiomyopathy.

**Methods:**

All TTR mutation carriers and ATTRm patients who underwent both ^123^I-MIBG and ^99m^Technetium-hydroxymethylene diphosphonate (^99m^Tc-HDP) scintigraphy were included. Cardiomyopathy was defined as NT-proBNP > 365 ng/L, and cardiac autonomic neuropathy as abnormal cardiovascular reflexes at autonomic function tests. Late ^123^I-MIBG heart-to-mediastinum ratio (HMR) < 2.0 or wash-out > 20%, and any cardiac ^99m^Tc-HDP uptake were considered as abnormal.

**Results:**

39 patients (13 carriers and 26 ATTRm patients) were included in this study. Patients with cardiomyopathy, with or without cardiac autonomic neuropathy, had lower late HMR than similar patients without cardiomyopathy [median 1.1 (range 1.0-1.5) and 1.5(1.2-2.6) vs 2.4 (1.4-3.8) and 2.5 (1.5-3.7), respectively, *P *< 0.001]. Late HMR and wash-out (inversely) correlated with NT-proBNP *r* = − 0.652 (*P *< 0.001) and *r* = 0.756 (*P *< 0.001), respectively. Furthermore, late HMR and wash-out (inversely) correlated with cardiac ^99m^Tc-HDP uptake *r* = − 0.663 (*P *< 0.001) and *r* = 0.617 (*P *< 0.001), respectively.

**Conclusion:**

In case of heart failure, ^123^I-MIBG scintigraphy reflects cardiomyopathy rather than cardiac autonomic neuropathy in ATTRm patients and TTR mutation carriers. ^123^I-MIBG scintigraphy may already be abnormal before any cardiac bone tracer uptake is visible.

**Electronic supplementary material:**

The online version of this article (10.1007/s12350-018-01477-y) contains supplementary material, which is available to authorized users.

## Introduction

Amyloidosis comprises a group of diseases, all characterized by the deposition of insoluble amyloid fibrils derived from soluble misfolded proteins. These deposits change the structure of tissues resulting in dysfunction of several organs, including the heart.^1^ Cardiac deposits cause symptoms of restrictive cardiomyopathy and cardiac autonomic neuropathy, when the myocardium and the cardiac conductive system, respectively, are infiltrated.^2^ Hereditary transthyretin (TTR)-derived amyloidosis (mutant transthyretin, ATTRm) and senile transthyretin-derived amyloidosis (wild-type transthyretin, ATTRwt) are the two types of systemic amyloidosis that originate from TTR^1^,^3^ and both are associated with cardiac involvement.^4^,^5^ Endomyocardial biopsy is currently the gold standard for diagnosing cardiac amyloidosis,^6^^–^8 but this procedure is time consuming with an increased risk of complications, such as arrhythmias, puncture of central arteries, pneumothorax, and perforation with pericardial tamponade.^9^

While searching for noninvasive alternatives, N-terminal pro-B-type natriuretic peptide (NT-proBNP)^10^^–^12 and autonomic function tests became of value as markers of cardiomyopathy and cardiac autonomic neuropathy, respectively.^13^,^14^ However, both noninvasive alternatives have limitations: loss of renal function and arrhythmias may cause elevated NT-proBNP levels irrespective of the presence of cardiomyopathy,^15^ and autonomic function tests mainly assess cardiac parasympathetic function.^13^,^14^

Bone seeking tracers, such as ^99m^Technetium-pyrophosphate (^99m^Tc-PYP),^16^^99m^Technetium-hydroxymethylene diphosphonate (^99m^Tc-HDP),^17^,^18^ and ^99m^Technetium-3,3-diphosphono-1,2-propanocarboxylic acid (^99m^Tc-DPD)^19^,^20^ are used to differentiate immunoglobulin light-chain amyloidosis (AL) from ATTR.^21^,^22^ The reason of positive tracer uptake by cardiac ATTR amyloid is not really understood. Since there is a higher density of micro-calcifications and fewer macrophages in ATTR compared with AL,^23^ bone scintigraphy probably shows a higher myocardial uptake in patients with ATTR.^8^,^21^,^24^,^25^ The question remains, however, to which degree positive cardiac uptake of a bone scintigraphy reflects the presence and severity of cardiomyopathy.

Additional ^123^Iodine-metaiodobenzylguanidine (^123^I-MIBG) scintigraphy is used for the visualization and quantitation of the sympathetic innervation of the heart,^26^ reflecting the sympathetic tone. Patients with cardiac amyloidosis tend to show a decreased heart-to-mediastinum ratio (HMR) and a higher wash-out rate, compared to healthy subjects.^27^ This indicates an impaired ^123^I-MIBG uptake and thereby an impairment of the cardiac sympathetic function.^26^^–^29 However, it remains unclear if an impaired cardiac sympathetic innervation in ATTRm patients reflects cardiac autonomic neuropathy or cardiomyopathy.

So far, little is known about the results of the combined nuclear scans in comparison with the severity of the disease, the degree of cardiac involvement, and the correlation with other markers for cardiomyopathy and autonomic neuropathy of the heart. All patients in our center with an amyloidogenic TTR mutation were studied. Not only patients with proven amyloid were studied, but also carriers of the mutation in whom prefibrillar aggregates already may be present.^30^ Therefore, the aim of this study was to

1. Gain insight of whether nuclear imaging modalities in patients with ATTRm amyloidosis and carriers of a mutation reflect cardiomyopathy or cardiac autonomic neuropathy, or both, and to
2. Gain insight into the utility of both bone scintigraphy and ^123^I-MIBG scintigraphy in early detection of cardiomyopathy or neuropathy in these patients.

## Methods

### Patients

In total, 40 patients with an amyloidogenic TTR mutation underwent both ^123^I-MIBG scintigraphy and ^99m^Tc-HDP scintigraphy as general work-up for newly diagnosed ATTRm patients in the University Medical Center Groningen (UMCG) between November 2007 and October 2017. The amyloidogenic TTR mutation was found by genetic testing of nucleated blood cells using DNA sequencing.^6^,^7^

All patients underwent an abdominal subcutaneous fat biopsy, autonomic function tests, neurologic tests, 12-leads electrocardiography (ECG), dynamic electrocardiography (Holter investigation), echocardiography, laboratory tests (creatinine, NT-proBNP and troponin T) and ^99m^Tc-HDP scintigraphy within 1 year before or after the ^123^I-MIBG scintigraphy.

Patient history retrieved from the electronic patient chart, physical examination, and the function tests mentioned above were all used to assess the presence of neuropathy (small fiber neuropathy, large fiber neuropathy and autonomic neuropathy). The presence of cardiomyopathy was defined as NT-proBNP > 365 ng/L,^10^ not explained by renal failure. Cardiac autonomic neuropathy was defined as the presence of abnormal cardiovascular reflexes at autonomic function tests, especially bedside maneuvers.

According to the local Dutch regulations for retrospective observational studies, formal ethical approval was not required. A waiver was acquired by our local ethics committee on October 1, 2017.

### ^123^I-MIBG Scintigraphy

^123^I-MIBG scintigraphy was performed as described in our previous study^27^: after blockade of thyroid uptake by iodine potassium iodide, patients were intravenously injected with 5.0 mCi (185 MBq) ^123^I-MIBG. Planar images of the thorax were made after 15 minutes and 4 hours after injection to measure, respectively, the early and late HMR.^27^ HMR was determined by the counts in a manually drawn region-of-interest (ROI) along the contour of the left ventricle, divided by the counts in a fixed ROI in the upper mediastinum. The cardiac wash-out rate was defined as a change in percentage of the activity ratio, calculated as follows:

[(HMR_early_−HMR_late_)/HMR_early_] × 100%.^26^^–^30 Due to the use of a medium energy collimator, either late HMR < 2.0 or wash-out rate > 20% were considered as abnormal ^123^I-MIBG parameters, and hence suggestive for impaired sympathetic cardiac innervation.^31^^–^33

### ^99m^Tc-HDP Scintigraphy

^99m^Tc-HDP scintigraphy was performed as described in our previous study^18^: patients were intravenously injected with 20 (750 MBq; up to January 1st 2016) or 14 (500 MBq; from January 1st 2016 onward) mCi ^99m^Tc-HDP and 3 hours after tracer administration, planar whole body images with single photon emission computed tomography (SPECT) computed tomography (CT) of the thorax including the heart were performed.^18^ The visual scoring of the cardiac uptake was scored as follows: (0) absent cardiac uptake; (1) mild cardiac uptake (less than bone); (2) moderate cardiac uptake (equal to bone); (3) high cardiac uptake (greater than bone).^18^ Any cardiac uptake was considered as suggestive for cardiac amyloidosis, since any ^99m^Tc-HDP uptake is highly sensitive for early stage cardiac amyloidosis in ATTRm patients.^17^^–^20

### Neurologic and Autonomic Function Tests

Quantitative sensory testing (QST) was performed to detect small fiber neuropathy, and electromyography (EMG) and nerve conduction velocity (NCV) testing were used to detect large fiber neuropathy. The presence of carpal tunnel syndrome was not considered to be a manifestation of polyneuropathy.

The autonomic function tests include bedside maneuvers (deep breathing test, Valsalva maneuver, isometric handgrip test, and heart rate and blood pressure response to standing up),^13^,^14^ and sympathetic skin response. The presence of autonomic neuropathy was defined as the presence of signs of disturbed cardiovascular reflexes (signs of orthostatic hypotension, and abnormal bedside maneuvers), disturbed gastrointestinal reflexes (symptoms of gastric emptying disorder, intestinal obstruction, diarrhea, constipation, and incontinence), disturbed urogenital reflexes (symptoms of bladder dysfunction, incontinence, and erectile dysfunction), or abnormal sympathetic skin response.^34^,^35^

### Echocardiographic Examination

Using the standard echocardiographic techniques, the following variables were assessed: left ventricular ejection fraction (LVEF), granular sparkling, and interventricular wall thickness at end-diastole (IVSd). Echocardiographic amyloidosis was defined as the presence of granular sparkling of the ventricular myocardium and a mean IVSd > 12 mm in the absence of potential causes of left ventricular hypertrophy, such as hypertension.^25^,^35^^–^37

### Electrocardiographic Investigation

The presence of conduction system disease (intraventricular conduction delay, left bundle branch block, and right bundle branch block), and arrhythmias, pseudo-infarction, and low QRS voltage on 12-leads ECG were considered suggestive for cardiac amyloidosis.^6^,^37^^–^39

### Study Design

After inclusion, the patients were divided into four groups based on NT-proBNP and autonomic function tests (considered as gold standards for cardiomyopathy and neuropathy, respectively): (C−N−) no signs of cardiac autonomic neuropathy and cardiomyopathy; (C−N+) no signs of cardiomyopathy, however positive for autonomic neuropathy of the heart; (C+N−) signs of cardiomyopathy, without signs of autonomic neuropathy of the heart; (C+N+) signs of both cardiac autonomic neuropathy and cardiomyopathy. Afterward, the database was reorganized, dividing all patients into four subgroups based on findings on imaging only: (M−B−) both normal ^123^I-MIBG and ^99m^Tc-HDP scintigraphy; (M+B−) abnormal ^123^I-MIBG scintigraphy and normal ^99m^Tc-HDP scintigraphy; (M−B+) normal ^123^I-MIBG scintigraphy and abnormal ^99m^Tc-HDP scintigraphy; and (M+B+) both abnormal ^123^I-MIBG and ^99m^Tc-HDP scintigraphy.

### Statistical Analysis

For statistical analyses, IBM SPSS Statistics version 25.0 was used. Patient characteristics were displayed as number (*n*), mean ± standard deviation (SD) when normally distributed, and median (range) when not normally distributed. The differences between the groups were tested using the independent *t* test (for normally distributed continuous variables), the Mann-Whitney *U* test (for not normally distributed continuous variables), and the Fisher’s exact, and Chi-square test (for categorical variables). Correlations were tested using the Pearson’s correlation test for continuous variables, and the Spearman’s rho correlation test for correlations between continuous and ordinal variables. *P*-values < 0.05 were considered statistically significant.

## Results

### Patient Characteristics

Since one patient had to be excluded due to missing autonomic function tests, 39 patients (21 men and 18 women) were included for statistical analysis. The overall mean age at the time of ^123^I-MIBG scintigraphy was 51 ± 14 years. 48% of the patients were carriers of the Val30Met mutation. Non-Val30Met mutations found were Tyr114Cys (18%), Glu89Lys (10%), Val71Ala (7.5%), Ser23Asn (5.0%), Val94Ala (2.5%), Gly47Glu (2.5%), Ala45Gly (2.5%), Val122del (2.5%), and Val122Ile (2.5%). Twenty-six (67%) patients had histologically proven amyloidosis. Of the 26 patients with amyloidosis, 8 had signs of cardiac involvement, 11 had signs of cardiac autonomic neuropathy, 13 had cardiac ^99m^Tc-HDP uptake, and 16 had an abnormal ^123^I-MIBG scintigraphy. Of the 13 carriers, 1 had signs of cardiac involvement, 2 had signs of cardiac autonomic neuropathy, 3 had cardiac ^99m^Tc-HDP uptake, and 2 had an abnormal ^123^I-MIBG scintigraphy.

Patients were divided into four different subgroups based on the presence of cardiomyopathy (C) and cardiac autonomic neuropathy (N): 22 patients were included in group C−N−, 8 in group C−N+, 4 in group C+N−, and 5 patients were included in group C+N+. There was no significant difference in gender, age at diagnosis, and age at ^123^I-MIBG scintigraphy between the patient groups. The duration of amyloidosis, time from positive DNA test to ^123^I-MIBG scintigraphy, was longer in patients from group C−N− and C+N− (respectively, 30 and 52 months) compared to patients from group C−N+ and C+N+ (respectively, 10 and 7.0 months), although not significant (*P *= 0.353). Twelve (60%) patients in group C−N− had histologically proven amyloidosis vs 6 (75%) patients in group C−N+, 3 (75%) in group C+N−, and all 5 patients in group C+N+ (Table 1).

**Table 1:** Patient characteristics for patient groups based on cardiac autonomic neuropathy and cardiomyopathy

Characteristic	Group C−N− (*n *= *22*)	Group C−N+ (*n *=* 8*)	Group C + N− (*n *=* 4*)	Group C+N+ (*n *=* 5*)	*P*-value
Gender (*n*)					0.638
Male	11	4	2	4	
Female	11	4	2	1	
Age at diagnosis (y), median (range)	41 (22–64)	46 (29–69)	61 (32–78)	50 (35–73)	0.315
Age at ^123^I-MIBG scan (y), median (range)	45 (26–69)	58 (30–69)	65 (52–78)	60 (36–73)	0.129
Duration (m), median (range)	30 (1.0–172)	10 (2.0–295)	52 (3.0–268)	7.0 (0.0–118)	0.353
Amyloid biopsy (*n*)					0.120
Positive	12	6	3	5	
Negative	10	2	1	0	
^123^I-MIBG parameters, median (range)					
Late HMR	2.5 (1.5–3.7)	2.4 (1.4–3.8)	1.5 (1.2–2.6)	1.1 (1.0–1.5)	0.002
Wash-out rate (%)	2.5 (− 17–22)	5.0 (− 25–20)	26 (11–34)	26 (15–38)	0.001
Abnormal ^123^I-MIBG parameters (*n*)	7	3	3	5	0.011
^99m^Tc-HDP findings (*n*)					0.002
Absent cardiac uptake	17	4	2	0	
Mild cardiac uptake	3	1	0	0	
Moderate cardiac uptake	0	3	1	3	
High cardiac uptake	2	0	1	2	
Laboratory results, median (range)					
Creatinine (umol/L)	76 (53–118)	76 (58-84)	83 (66–99)	69 (64–103)	0.876
NT-proBNP (ng/L)	54 (16–125)	207 (8.0–287)	1052 (368–2422)	1572 (368–6222)	< 0.001
Troponin T (ng/L)	4.5 (3.0–24)	4.0 (< 0.01–64)	25 (3.0–55)	37 (25–64)	0.008

Group C−N− = no cardiac autonomic neuropathy and cardiomyopathy, group C−N+=only cardiac autonomic neuropathy, group C+N− = only cardiomyopathy, group C+N+= both cardiac autonomic neuropathy and cardiomyopathy

^*123*^*I-MIBG*, ^123^Iodine-metaiodobenzylguanide; ^*99m*^*Tc-HDP*, ^99m^Technetium-hydroxymethylene diphosphonate; *HMR*, heart-to-mediastinum ratio; *m*, months; *n*, number; *y*, years

### Laboratory Results

**Figure 1:**
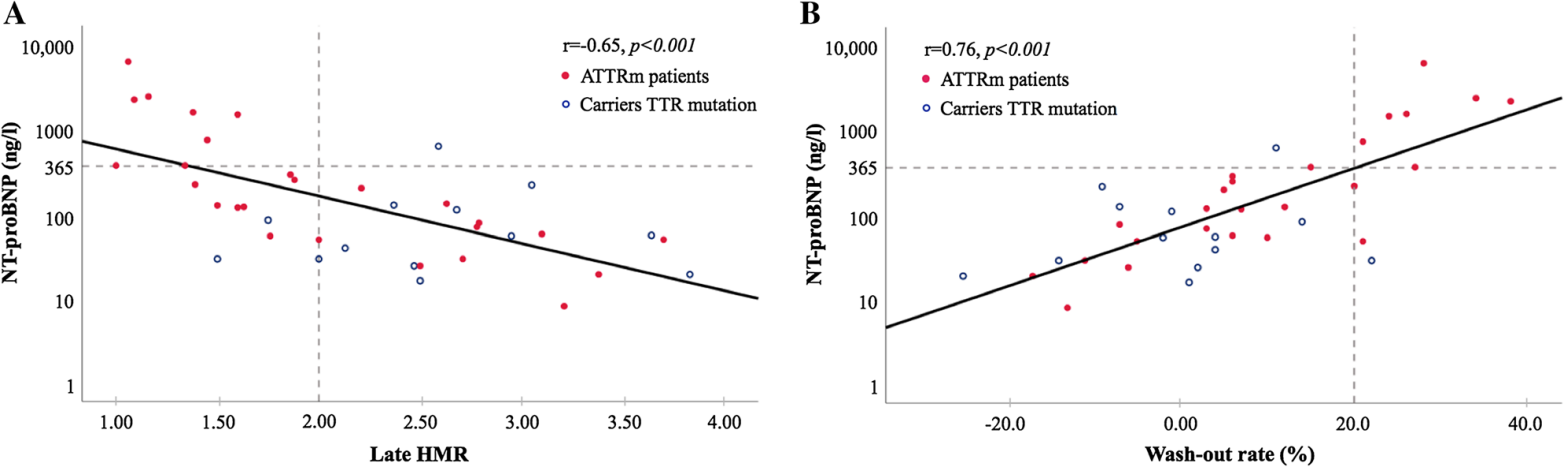
Correlation between log-transformed NT-proBNP and (**A**) late HMR (heart-to-mediastinum ratio, (**B**) ^123^I-MIBG (^123^Iodine-metaiobenzylguanide) wash-out rates. Cutoff values for late HMR and wash-out rate (vertical gray broken lines) and NT-proBNP (horizontal gray broken line). Linear regression (solid lines). Carriers TTR mutation (blue open circles) and ATTRm (hereditary transthyretin amyloidosis) patients (red filled circles)

Creatinine did not differ significantly between the patient groups. Median NT-proBNP was significantly higher in patients from groups C+N− and C+N+, 1052 (range 368-2422) and 1572 (368-6222), respectively, vs 54(16-125) for group C−N− and 207 (8.0-287) ng/L for group C−N+ (*P *< 0.001). Likewise, patients in groups C+N− and C+N+ had higher troponin T values compared to patients in groups C−N− and C−N+, 25 (3.0-55) and 37 (25-64) vs 4.5 (3.0-24) and 4.0 (< 0.01-64) ng/L, respectively (*P *= 0.008; Table 1). A negative correlation between NT-proBNP and late HMR was found (*r* = − 0.65, *P *< 0.001; Figure 1A). Patients with late HMR < 2.0 had higher NT-proBNP values compared to patients with normal late HMR. Figure 1B shows the positive correlation between NT-proBNP and ^123^I-MIBG wash-out rates (*r* = 0.76 *P *< 0.001). Patients with abnormal wash-out rates had higher NT-proBNP values and vice versa.

### ECG and Echocardiography

LVEF on echocardiography did not differ significantly between the four groups (*P *= 0.875). All patients in group C+N+ had signs of granular sparkling on echocardiography vs 1 patient in group C−N−, 1 in patient group C−N+, and 3 patients in group C+N− (*P *< 0.001). Median IVSd was significantly higher in patients from group C+N+ compared to patients from the other patient groups: 17 (10-23) vs 9.0 (6.0-13)mm for patient group C−N−, 10 (6.0-11)mm for group C−N+ , and 12 (8.0-16)mm for patients in group C+N−, respectively (*P *= 0.013). All patients from groups C+N− and C+N+ had signs of cardiac amyloidosis on ECG vs 11 patients from group C-N- and 5 patients from patient group C−N + (*P *= 0.042). Overall, signs of conduction system disease were mostly found, and 4 patients had multiple signs of cardiac amyloidosis on ECG (Table 2).

**Table 2:** Echo- and electrocardiographic findings

	Group C−N− (*n *=* 22*)	Group C−N+ (*n *=* 8*)	Group C+N− (*n *=* 4*)	Group C+N+ (*n *=* 5*)	*P*-value
Echocardiographic findings					
LVEF (%), median (range)	55 (54–60)	55 (50–65)	55 (50–60)	60 (38–60)	0.875
Sparkling (*n*)	1	1	3	5	< 0.001
IVSd (mm), median (range)	9.0 (6.0–13)	10 (6.0–11)	12 (8.0−16)	17 (10−23)	0.013
ECG findings					0.042
No pathological signs	11	3	0	0	
Conduction system disease	3	3	1	1	
Arrhythmia	3	1	1	0	
Pseudo-infarction	3	1	0	3	
Multiple	1	0	2	1	

Group C−N− = no cardiac autonomic neuropathy and cardiomyopathy, group C−N+=only cardiac autonomic neuropathy, group C+N− = only cardiomyopathy, group C+N+ = both cardiac autonomic neuropathy and cardiomyopathy

*ECG*, electrocardiography; *LVEF*, left ventricular ejection fraction; *IVSd*, interventricular wall thickness at end-diastole; *mm*, millimeter; *n*, number

### ^123^I-MIBG and ^99m^Tc-HDP Scintigraphy Findings Related to Cardiomyopathy and Neuropathy

Late HMR was significantly lower in patients from groups C + N- and C+N+ (respectively, 1.5 (1.2-2.6) and 1.1 (1.0-1.5)) compared to patients from groups C−N− and C−N+ (respectively, 2.5 (1.5-3.7) and 2.4 (1.4-3.8), *P *= 0.002), and the wash-out rates were significantly higher in these patient groups, respectively, 26 (11-34)% in group C+N− and 26 (15-38)% in group C+N+ vs 2.5 (− 17 to 22)% in group C−N− and 5.0 (− 25 to 20)% in group C−N+ (*P *= 0.001). Patients in groups C+N− and C+N + had more often abnormal ^123^I-MIBG parameters: 3 (75%) in group C+N− and 5 (100%) in group C+N+ vs 7 (32%) in group C−N− and 3 (38%) in group C−N+ (*P *= 0.011). All patients in group C+N+ had cardiac uptake on ^99m^Tc-HDP scintigraphy (3 had moderate uptake, and 2 had high uptake) vs 5 patients in group C−N− (3 had mild uptake, and 2 had high uptake), 4 in group C−N+ (1 had mild uptake, and 3 had moderate cardiac uptake), and 2 patients in group C+N− (1 had moderate uptake, and 1 had high uptake, *P *= 0.002; Table 1).

### Correlation Between ^123^I-MIBG and ^99m^Tc-HDP Scintigraphy

**Figure 2:**
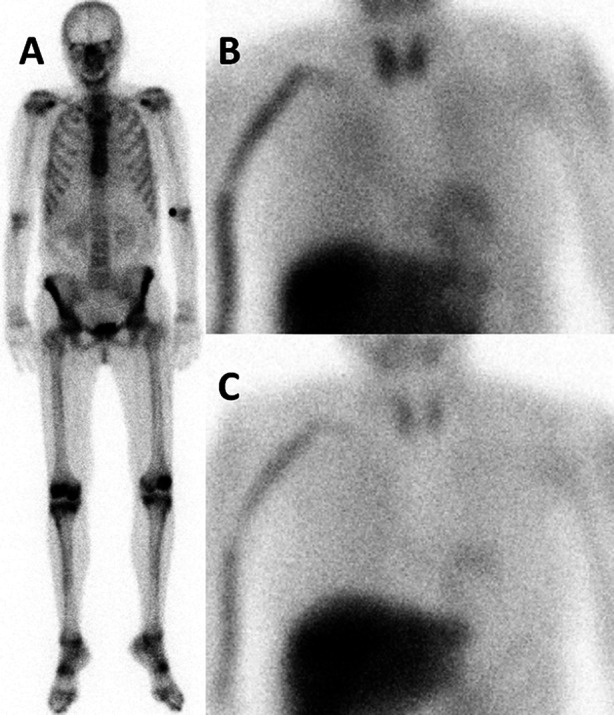
(**A**) Planar anterior view of ^99m^Tc-HDP (^99m^Technetium-hydroxymethylene diphosphonate) scintigraphy in a middle-aged female patient with ATTRm (mutant transthyretin amyloidosis), showing physiological tracer distribution in the bones and normal excretion through the urinary tract, but no cardiac tracer accumulation. Anterior views of (**B**) early time point and (**C**) late time point ^123^I-MIBG (^123^Iodine-metaiodobenzylguanide) scintigraphy, showing a decrease in cardiac ^123^I-MIBG HMR (heart-to-mediastinum ratio; decreasing from 1.70 to 1.50, respectively, normal value in our laboratory for late HMR 2.00)

Based on the above described results, patients were re-divided into four groups based on imaging findings: 18 patients were included in group M-B-, 5 in group M+B− (Figure 2), 3 in group M−B+, and 13 patients were included in group M+B+.

**Figure 3:**
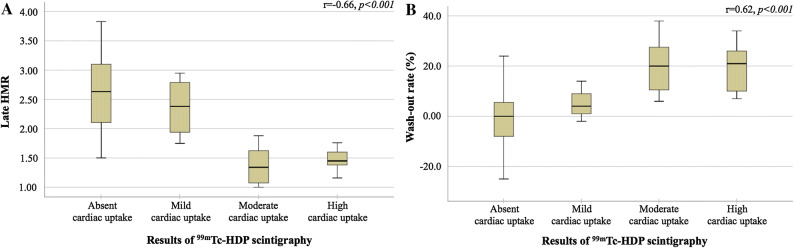
(**A**) Late HMR (heart-to-mediastinum ratio) and (**B**) wash-out rates for all categories of cardiac ^99m^Tc-HDP (^99m^Technetium-hydroxymethylene diphosphonate) uptake

There was an inverse correlation between late HMR and cardiac uptake on ^99m^Tc-HDP scintigraphy as shown in Figure 3A (*r* = − 0.66*, P *< 0.001). On the contrary, a positive correlation between ^123^I-MIBG wash-out rates and cardiac uptake on ^99m^Tc-HDP scintigraphy was found (*r* = 0.62, *P *< 0.001; Figure 3B).

Group M+B+ contained statistically significantly more men than group M−B− (respectively, 10 and 6, *P *= 0.017). Late HMR was significantly lower in patients from group M+B+ compared to group M−B− (1.4(1.0-1.9) vs 2.7(2.0-3.8), *P *< 0.001) and the wash-out rates were significantly higher in this patient group, respectively, 20 (6.0-38) vs − 5.5 (− 25 to 11) % (*P *< 0.001). In group M+B+ , one patient showed mild uptake, 7 showed moderate uptake and 5 patients showed high cardiac uptake on ^99m^Tc-HDP scintigraphy. Both NT-proBNP and troponin T were significantly higher in patients from group M+B+ compared to group M−B−: 368 (54-6222) vs 97 (8.0-623) ng/L (*P *= 0.001), and 28 (5.0-64) vs 3.0 (3.0-14) ng/L (*P *< 0.001; Table 3).

**Table 3:** Patient characteristics for subgroups based on ^123^I-MIBG and ^99m^Tc-HDP findings

Characteristic	Group M−B− (*n *= *18*)	Group M+B− (*n *=* 5*)	Group M−B+ (*n *=* 3*)	Group M+B+ (*n *=* 13*)	*P*-value*
Gender (*n*)					0.017
Male	6	2	3	10	
Female	12	3	0	3	
Amyloid biopsy (*n*)					0.032
Positive	9	4	1	12	
Negative	9	1	2	1	
^123^I-MIBG parameters, median (range)					
Late HMR	2.7 (2.0–3.8)	1.6 (1.5–2.0)	2.5 (2.1–3.0)	1.4 (1.0–1.9)	< 0.001
Wash-out rate (%)	− 5.5 (− 25–11)	21 (3.0–24)	1.0 (− 2.0–4.0)	20 (6.0–38)	< 0.001
^99m^Tc-HDP findings (*n*)					< 0.001
Absent cardiac uptake	18	5	0	0	
Mild cardiac uptake	0	0	3	1	
Moderate cardiac uptake	0	0	0	7	
High cardiac uptake	0	0	0	5	
Laboratory results, median (range)					
NT-proBNP (ng/L)	97 (8.0–623)	119 (29–1481)	5.0 (39–130)	368 (54–6222)	0.001
Troponin T (ng/L)	3.0 (3.0–14)	4.0 (4.0–8.0)	5.0 (< 0.01–10)	28 (5.0–64)	< 0.001

Group M−B− = normal ^123^I-MIBG and ^99m^Tc-HDP scintigraphy, group M+B− = abnormal ^123^I-MIBG scintigraphy, group M−B+=abnormal ^99m^Tc-HDP scintigraphy

Group M+B+= abnormal ^123^I-MIBG and ^99m^Tc-HDP scintigraphy

^*123*^*I-MIBG*, ^123^Iodine-metaiodobenzylguanide; ^*99m*^*Tc-HDP*, ^99m^Technetium-hydroxymethylene diphosphonate; *HMR*, heart-to-mediastinum ratio; *n*, number

*P-values between groups M-B- and M+B+

### Development of Neuropathy and Cardiomyopathy

Table 4 summarizes the presence of carpal tunnel syndrome, small and large fiber neuropathy, autonomic neuropathy, and cardiomyopathy in all four patient groups. Besides the presence of amyloid, no other causes for peripheral and autonomic neuropathy, such as diabetes mellitus, were identified in any of the patients. Significantly more patients from group M+B+ had signs of large fiber neuropathy, autonomic neuropathy and cardiomyopathy compared to patient group M−B−: 12 vs one patient (*P *< 0.001), 12 vs 10 patients (*P *= 0.045), and 11 vs one patient in group M−B−, respectively (*P *= 0.004). No significant differences in carpal tunnel syndrome and small fiber neuropathy were found between groups M−B− and M+B+ (respectively, *P *= 0.069, and *P *= 0.060).

**Table 4:** Presence of neuropathy and cardiomyopathy

	Group M−B− (*n *=18)	Group M+B− (*n *=5)	Group M−B+ (*n *=3)	Group M+B+ (*n *=13)	*P*-value*
Carpal tunnel syndrome (*n*)	4	1	1	7	0.069
Small fiber neuropathy (*n*)	13	4	3	12	0.060
Large fiber neuropathy (*n*)	4	4	3	12	< 0.001
Autonomic neuropathy (*n*)	10	4	3	12	0.045
Cardiomyopathy (*n*)	1	1	1	11	0.004

Group M−B− = normal ^123^I-MIBG and ^99m^Tc-HDP scintigraphy, group M+B− = abnormal ^123^I-MIBG scintigraphy, group M−B+=abnormal ^99m^Tc-HDP scintigraphy

Group M+B+= abnormal ^123^I-MIBG and ^99m^Tc-HDP scintigraphy

*n*, number

*P-values between groups M−B− and M+B+

**Figure 4:**
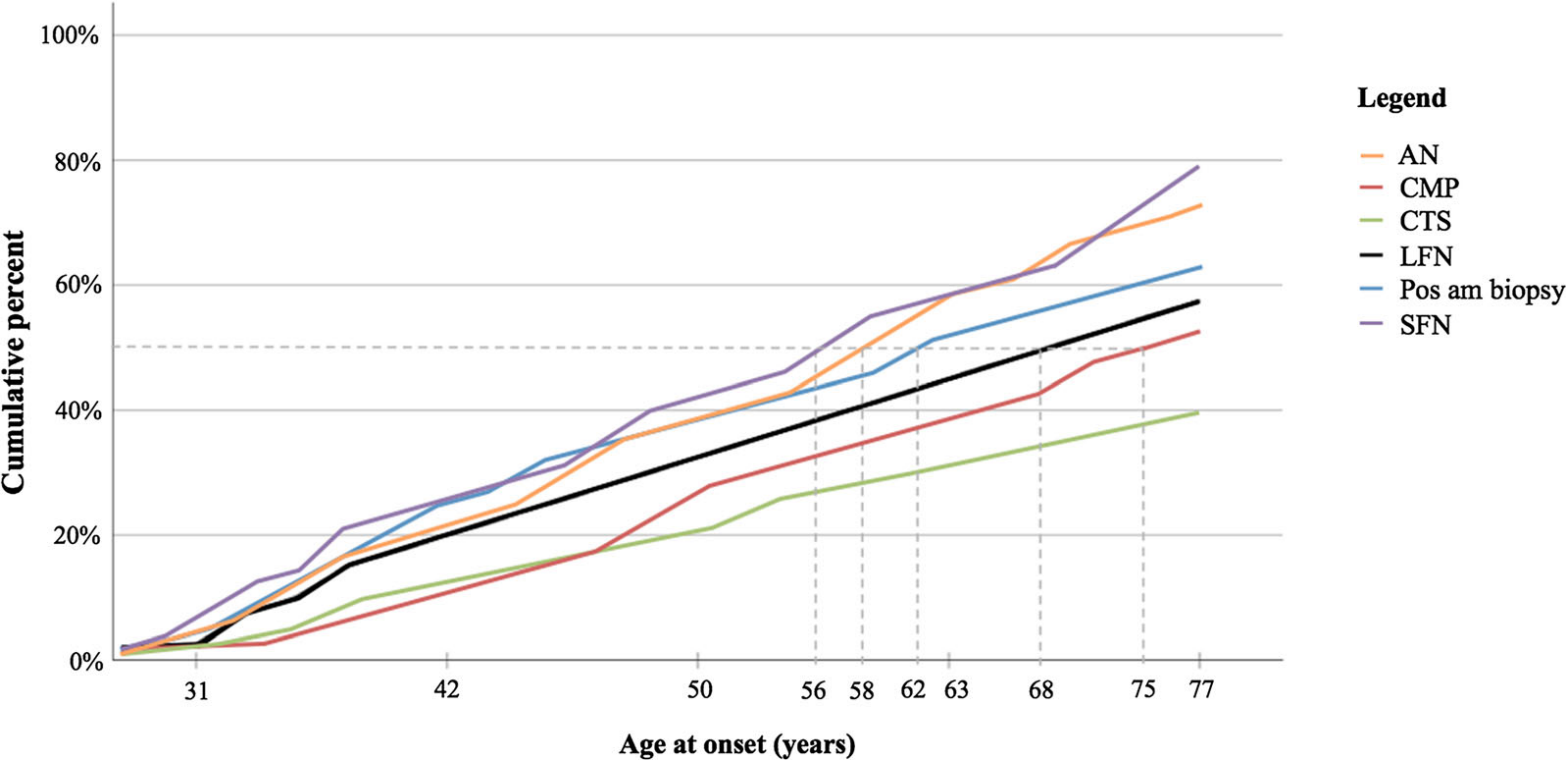
Cumulative incidence of symptoms for increasing age at time of analysis. Age at which half of the patients (gray broken line) had autonomic neuropathy (AN, orange line), cardiomyopathy (CMP, red line), carpal tunnel syndrome (CTS, green line), large fiber neuropathy (LFN, black line), positive amyloid biopsy (Pos am biopsy, blue line), and small fiber neuropathy (SFN, purple line)

Figure 4 shows the age at which patients, cumulatively arranged by age, already had developed signs of neuropathy (autonomic neuropathy, large fiber neuropathy, and small fiber neuropathy), carpal tunnel syndrome, cardiomyopathy, and positive amyloid biopsy. Half of the patients with a genetically proven mutation also had a positive amyloid biopsy at the age of 62. Autonomic neuropathy and small fiber neuropathy tend to manifest at an earlier age, respectively, at the ages of 58 and 56. On the contrary, large fiber neuropathy tends to manifest in advanced stages of the disease, at the age of 68. Signs of carpal tunnel syndrome and cardiomyopathy were not found in 50% of the patients.

## Discussion

Similar to cardiac ^99m^Tc-HDP uptake, abnormal findings on ^123^I-MIBG scintigraphy reflect cardiomyopathy and do not reflect cardiac autonomic neuropathy in case of heart failure. Furthermore, abnormal findings on ^123^I-MIBG scintigraphy precede cardiac uptake on ^99m^Tc-HDP scintigraphy.

The principle of autonomic dysfunction and interpretation of ^123^I-MIBG scintigraphy in ATTRm patients is rather difficult to understand.^40^,^41^ Sympathetic denervation and heart failure seem to coincide and contribute to each other, especially in advanced stages of the disease.^42^ However, a recent study of Piekarski et al showed that 48% of asymptomatic patients show abnormal ^123^I-MIBG findings whereas no cardiac uptake on ^99m^Tc-DPD scintigraphy is visible.^42^ This also suggests that cardiac sympathetic denervation precedes cardiac uptake on bone scintigraphy, even though in this study a different collimator was used to measure the ^123^I-MIBG uptake.

The NT-proBNP values have not been compared to the results of the ^123^I-MIBG scintigraphy before. An earlier study of our center assessed the diagnostic value of NT-proBNP for determining the cardiac involvement in ATTRm patients. The cutoff value > 365 ng/L used in the current study was found to have the highest specificity.^10^

The results from the current study underline that both cardiac bone seeking tracer accumulation and decreased late HMR on ^123^I-MIBG scintigraphy reflect cardiac involvement of amyloidosis. Furthermore, we found that signs of small fiber neuropathy and autonomic neuropathy are already present in early stages of the disease, before amyloid has been detected in a biopsy and before ^123^I-MIBG and ^99m^Tc-HDP scintigraphy are positive. It is possible that in these cases amyloid already may be present, but not yet detected in a biopsy because of sampling error. Another possibility is that prefibrillar TTR aggregates may be present already affecting the nerve function, as has been suggested in the study of Sousa et al^30^ Some studies found that patients with signs of either polyneuropathy or autonomic neuropathy have higher ^123^I-MIBG wash-out rates and lower late HMR.^28^,^42^

The current study has some limitations. In this study, bedside maneuvers, such as the deep breathing test, Valsalva maneuver, isometric handgrip test, and heart rate and blood pressure response to standing up, were used to determine the presence of cardiac autonomic neuropathy. Most bedside maneuvers, however, mainly test the parasympathetic function because it is believed that parasympathetic dysfunction precedes sympathetic neuropathy.^13^,^14^ Since we used bedside maneuvers to test the presence of autonomic neuropathy of the heart, it is possible that some patients with sympathetic dysfunction had abnormal ^123^I-MIBG parameters and normal autonomic function tests of the heart. Furthermore, besides neurologic and autonomic function tests, patient history retrieved from the electronic patient chart was used to determine the presence of neuropathy. Because of the retrospective nature of this study, this information is biased by the patient’s recall and the examiner’s questions. In addition, the number of patients included in this study is relatively low, although amyloidosis is a rare disease. As a result, the data analysis could be influenced by some missing cases, even though only a maximum of two parameters per patient group were missing. Although the statistics from the current study itself may have to be interpreted with caution, the results from this study are very much in line with recently reported findings in a cohort roughly twice the size of the current group.^42^

## New Knowledge Gained

The role of ^123^I-MIBG scintigraphy in the work-up of amyloidosis patients is up to present underestimated. Since ^123^I-MIBG scintigraphy is able to detect impaired cardiac sympathetic innervation before signs of cardiac ATTRm amyloidosis are visible on echocardiography and bone scintigraphy, and our results suggest that ^123^I-MIBG scintigraphy seems to reflect cardiomyopathy more than cardiac neuropathy in case of heart failure, we support a more prominent role for ^123^I-MIBG scintigraphy within the general work-up for newly diagnosed ATTRm amyloidosis patients. The documented important prognostic value of abnormal ^123^I-MIBG results^40^,^43^ may possibly be more related to it being a marker of ATTRm cardiomyopathy than a marker of cardiac autonomic neuropathy in case of heart failure.

## Conclusions

Late HMR and wash-out rate on ^123^I-MIBG scintigraphy reflect cardiomyopathy rather than cardiac autonomic neuropathy in ATTRm patients and carriers of a TTR mutation in case of heart failure. Since ^123^I-MIBG scintigraphy may detect cardiac amyloidosis before bone scintigraphy does, it supports a more prominent role of ^123^I-MIBG scintigraphy in the evaluation of cardiac amyloidosis.

## Electronic supplementary material

Below is the link to the electronic supplementary material.

Supplementary material 1 (PPTX 418 kb)

## Abbreviations

^123^I-MIBG: ^123^I-metaiodobenzylguanidine
^99m^Tc-HDP: ^99m^Technetium-hydroxymethylene diphosphonate
ATTRm: Hereditary transthyretin-derived amyloidosis, mutant transthyretin
ECG: Electrocardiography
HMR: Heart-to-mediastinum ratio
IVSd: Interventricular wall thickness at end-diastole
LVEF: Left ventricular ejection fraction
NT-proBNP: N-terminal pro-B-type natriuretic peptide
TTR: Transthyretin

## References

[1] Merlini G, Bellotti V (2003). Molecular mechanisms of amyloidosis. N Engl J Med.

[2] Goldstein DS (2016). Cardiac dysautonomia and surviva in hereditary transthyretin amyloidosis. J Am Coll Cardiol Imaging.

[3] Benson MD, Kincaid JC (2007). The molecular biology and clinical features of amyloid neuropathy. Muscle Nerve.

[4] Tuzovic M, Yang EH, Baas AS (2017). Cardiac amyloidosis: Diagnosis and treatment strategies. Curr Oncol Rep..

[5] Shah KB, Inoue Y, Mehra MR (2006). Amyloidosis and the heart. Amyloid Int J Exp Clin Investig.

[6] Mankad AK, Shah KB (2017). Transthyretin cardiac amyloidosis. Curr Cardiol Rep.

[7] Ruberg FL, Berk JL (2012). Transthyretin (TTR) cardiac amyloidosis. Circulation.

[8] Bokhari S, Shahzad R, Castaño A, Maurer MS (2014). Nuclear imaging modalities for cardiac amyloidosis. J Nucl Cardiol..

[9] Cooper LT, Baughman KL, Feldman AM (2007). The role of endomyocardial biopsy in the management of cardiovascular disease: A scientific statement from the American Heart Association, the American College of Cardiology, and the European Society of Cardiology. Circulation.

[10] Klaassen SHC, Tromp J, Nienhuis HLA (2017). Frequency of and prognostic significance of cardiac involvement at presentation in hereditary transthyretin-derived amyloidosis and the value of N-terminal Pro-B-type natriuretic peptide. Am J Cardiol.

[11] Damy T, Deux JF, Moutereau S (2013). Role of natriuretic peptide to predict cardiac abnormalities in patients with hereditary transthyretin amyloidosis. Amyloid.

[12] Suhr OB, Anan I, Backman C (2008). Do troponin and B-natriuretic peptide detect cardiomyopathy in transthyretin amyloidosis?. J Intern Med.

[13] Ewing DJ, Clarke BF (1982). Diagnosis and management of diabetic autonomic neuropathy. Br Med J (Clin Res Ed).

[14] Oei-Reyners AKL, Oei-Reyners AKL (2002). Cardiovascular tests. Cardiovascular autonomic function tests: Methodological considerations and clinical application.

[15] Ponikowski P, Voors AA, Anker SD (2016). 2016 ESC Guidelines for the diagnosis and treatment of acute and chronic heart failure. Eur Heart J.

[16] Bokhari S, Castaño A, Pozniakoff T, Deslisle S, Latif F, Maurer MS (2013). 99 mTc-pyrophosphate scintigraphy for differentiating light-chain cardiac amyloidosis from the transthyretin-related familial and senile cardiac amyloidoses. Circ Cardiovasc Imaging.

[17] Galat A, Rosso J, Guellich A (2015). Usefulness of ^99m^ Tc-HMDP scintigraphy for the etiologic diagnosis and prognosis of cardiac amyloidosis. Amyloid.

[18] Glaudemans AWJM, van Rheenen RWJ, Noordzij W (2014). Bone scintigraphy with 99m-technetium-hydroxymethylene diphosphonate allows early diagnosis of cardiac involvement in patients with transthyretin-derived systemic amyloidosis. Amyloid.

[19] Perugini E, Guidalotti PL, Salvi F (2005). Noninvasive etiologic diagnosis of cardiac amyloidosis using 99mTc-3,3-diphosphono-1,2-propanodicarboxylic acid scintigraphy. J Am Coll Cardiol.

[20] Rapezzi C, Quarta CC, Guidalotti PL (2011). Role of 99mTc-DPD scintigraphy in diagnosis and prognosis of hereditary transthyretin-related cardiac amyloidosis. J Am Coll Cardiol Imaging.

[21] Gillmore JD, Maurer MS, Falk RH (2016). Nonbiopsy diagnosis of cardiac transthyretin amyloidosis. Circulation.

[22] Noordzij W, Glaudemans AWJM, Slart RHJA, Dierckx RA, Hazenberg BPC (2012). Clinical use of differential nuclear medicine modalities in patients with ATTR amyloidosis. Amyloid.

[23] Stats MA, Stone JR (2016). Varying levels of small microcalcifications and macrophages in ATTR and AL cardiac amyloidosis: implications for utilizing nuclear medicine studies to subtype amyloidosis. Cardiovasc Pathol.

[24] Noordzij W, Slart R, Tio R (2015). Nuclear imaging of the autonomic nervous system in cardiac amyloidosis. Autonomic innervation of the heart: role of moleculare imaging.

[25] Noordzij W, Glaudemans AWJM, Rapezzi C (2015). Nuclear imaging for cardiac amyloidosis. Heart Fail Rev.

[26] Tanaka M, Hongo M, Kinoshita O (1997). Iodine-123 metaiodobenzylguanidine scintigraphic assessment of myocardial sympathetic innervation in patients with familial amyloid polyneuropathy. J Am Coll Cardiol.

[27] Noordzij W, Glaudemans AWJM, van Rheenen RWJ (2012). I-Labelled metaiodobenzylguanidine for the evaluation of cardiac sympathetic denervation in early stage amyloidosis. Eur J Nucl Med Mol Imaging.

[28] Delahaye N, Dinanian S, Slama MS (1999). Cardiac sympathetic denervation in familial amyloid polyneuropathy assessed by iodine-123 metaiodobenzylguanidine scintigraphy and heart rate variability. Eur J Nucl Med.

[29] Slart RHJA, Glaudemans AWJM, Hazenberg BPC, Noordzij W, Noordzij W (2017). Imaging cardiac innervation in amyloidosis. J Nucl Cardiol.

[30] Sousa MM, Cardoso I, Fernandes R, Guimarães A, Saraiva MJ (2001). Deposition of transthyretin in early stages of familial amyloidotic polyneuropathy: Evidence for toxicity of nonfibrillar aggregates. Am J Pathol.

[31] Flotats A, Carrió I, Agostini D (2010). Proposal for standardization of123I-metaiodobenzylguanidine (MIBG) cardiac sympathetic imaging by the EANM Cardiovascular Committee and the European Council of Nuclear Cardiology. Eur J Nucl Med Mol Imaging.

[32] Nakajima K, Okuda K, Yoshimura M (2014). Multicenter cross-calibration of I-123 metaiodobenzylguanidine heart-to-mediastinum ratios to overcome camera-collimator variations. J Nucl Cardiol.

[33] Inoue Y, Abe Y, Kikuchi K, Matsunaga K, Masuda R, Nishiyama K (2017). Correction of collimator-dependent differences in the heart-to-mediastinum ratio in123I-metaiodobenzylguanidine cardiac sympathetic imaging: Determination of conversion equations using point-source imaging. J Nucl Cardiol.

[34] Wang AK, Fealey RD, Gehrking TL, Low PA (2008). Patterns of neuropathy and autonomic failure in patients with amyloidosis. Mayo Clin Proc.

[35] Gertz MA, Comenzo R, Falk RH (2005). Definition of organ involvement and treatment response in immunoglobulin light chain amyloidosis (AL): A consensus opinion from the 10th International Symposium on Amyloid and Amyloidosis. Am J Hematol.

[36] Falk RH, Dubrey SW (2010). Amyloid heart disease. Prog Cardiovasc Dis.

[37] Siddiqi OK, Ruberg FL (2017). Cardiac amyloidosis: An update on pathophysiology, diagnosis, and treatment. Trends Cardiovasc Med.

[38] Dungu JN, Anderson LJ, Whelan CJ, Hawkins PN (2012). Cardiac transthyretin amyloidosis. Heart.

[39] Rapezzi C, Lorenzini M, Longhi S (2015). Cardiac amyloidosis: The great pretender. Heart Fail Rev.

[40] Algalarrondo V, Antonini T, Théaudin M (2016). Cardiac dysautonomia predicts long-term survival in hereditary transthyretin amyloidosis after liver transplantation. J Am Coll Cardiol Imaging.

[41] Delahaye D, Le Guludec D, Dinanian S (2001). Myocardial muscarinic receptor upregulation and normal response to isoproterenol in denervated hearts by familial amyloid polyneuropathy. Circulation.

[42] Piekarski E, Chequer R, Algalarrondo V (2018). Cardiac denervation evidenced by MIBG occurs earlier than amyloid deposits detection by diphosphonate scintigraphy in TTR mutation carriers. Eur J Nucl Med Mol Imaging.

[43] Coutinho MC, Cortez-Dias N, Cantinho G (2013). Reduced myocardial 123-iodine metaiodobenzylguanidine uptake: a prognostic marker in familial amyloid polyneuropathy. Circ Cardiovasc Imaging.

