# Supplementary material for: Imaging cardiac innervation in hereditary transthyretin (ATTRm) amyloidosis: A marker for neuropathy or cardiomyopathy in case of heart failure?
Source: J Nucl Cardiol. 2018 Oct 29;27(5):1774–84. doi: 10.1007/s12350-018-01477-y (PMC7599160; doi:10.1007/s12350-018-01477-y)
Supplement: Supplementary file 1 — Supplementary material 1 (PPTX 418 kb) [file 12350_2018_1477_MOESM1_ESM.pptx]

## Slide 1
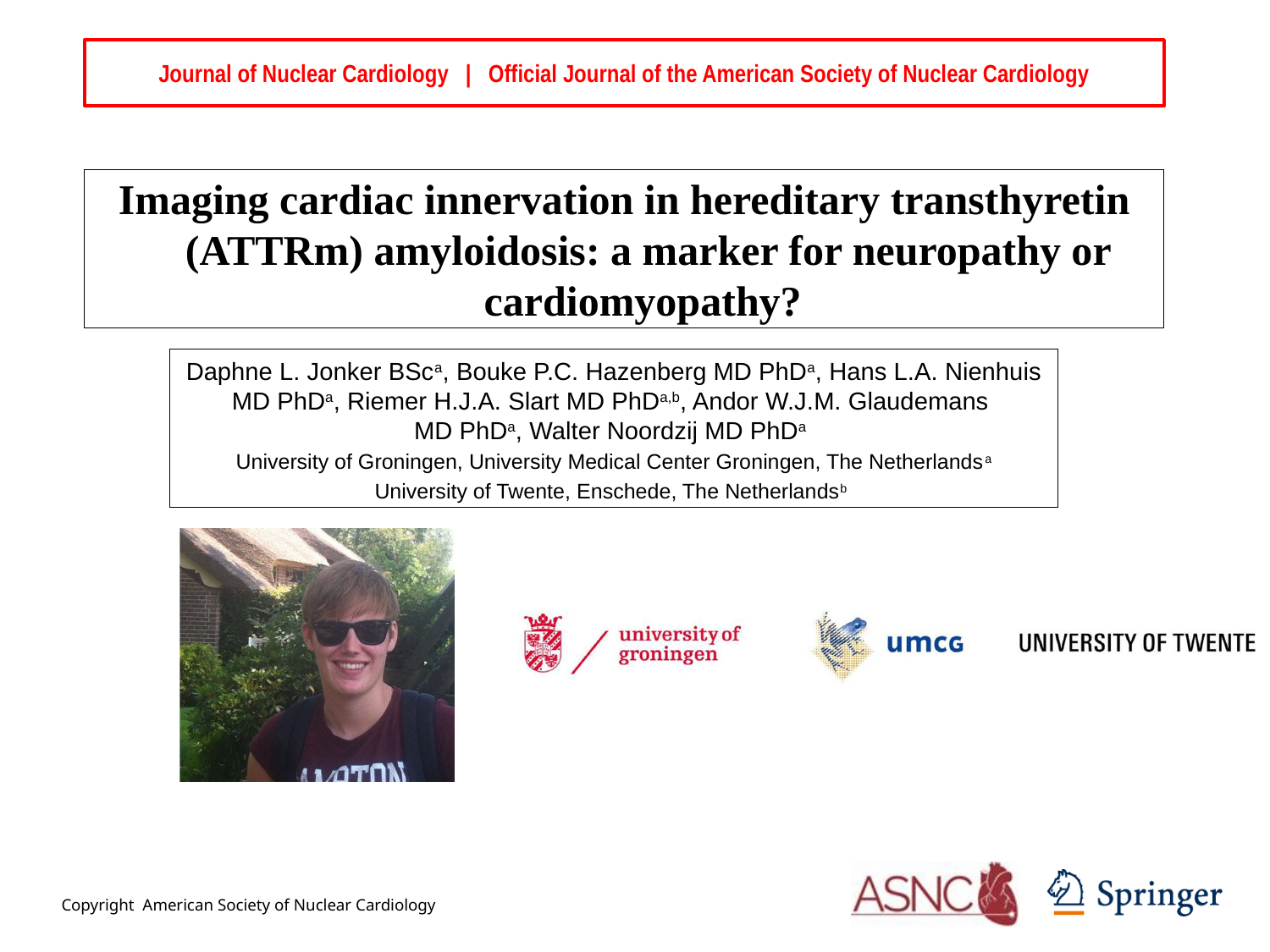

Journal of Nuclear Cardiology | Official Journal of the American Society of Nuclear Cardiology
# Imaging cardiac innervation in hereditary transthyretin (ATTRm) amyloidosis: a marker for neuropathy or cardiomyopathy?
Daphne L. Jonker BSca, Bouke P.C. Hazenberg MD PhDa, Hans L.A. Nienhuis MD PhDa, Riemer H.J.A. Slart MD PhDa,b, Andor W.J.M. Glaudemans
MD PhDa, Walter Noordzij MD PhDa
University of Groningen, University Medical Center Groningen, The Netherlandsa
University of Twente, Enschede, The Netherlandsb
Institution
Picture/Logo
Optional
Copyright American Society of Nuclear Cardiology

## Slide 2
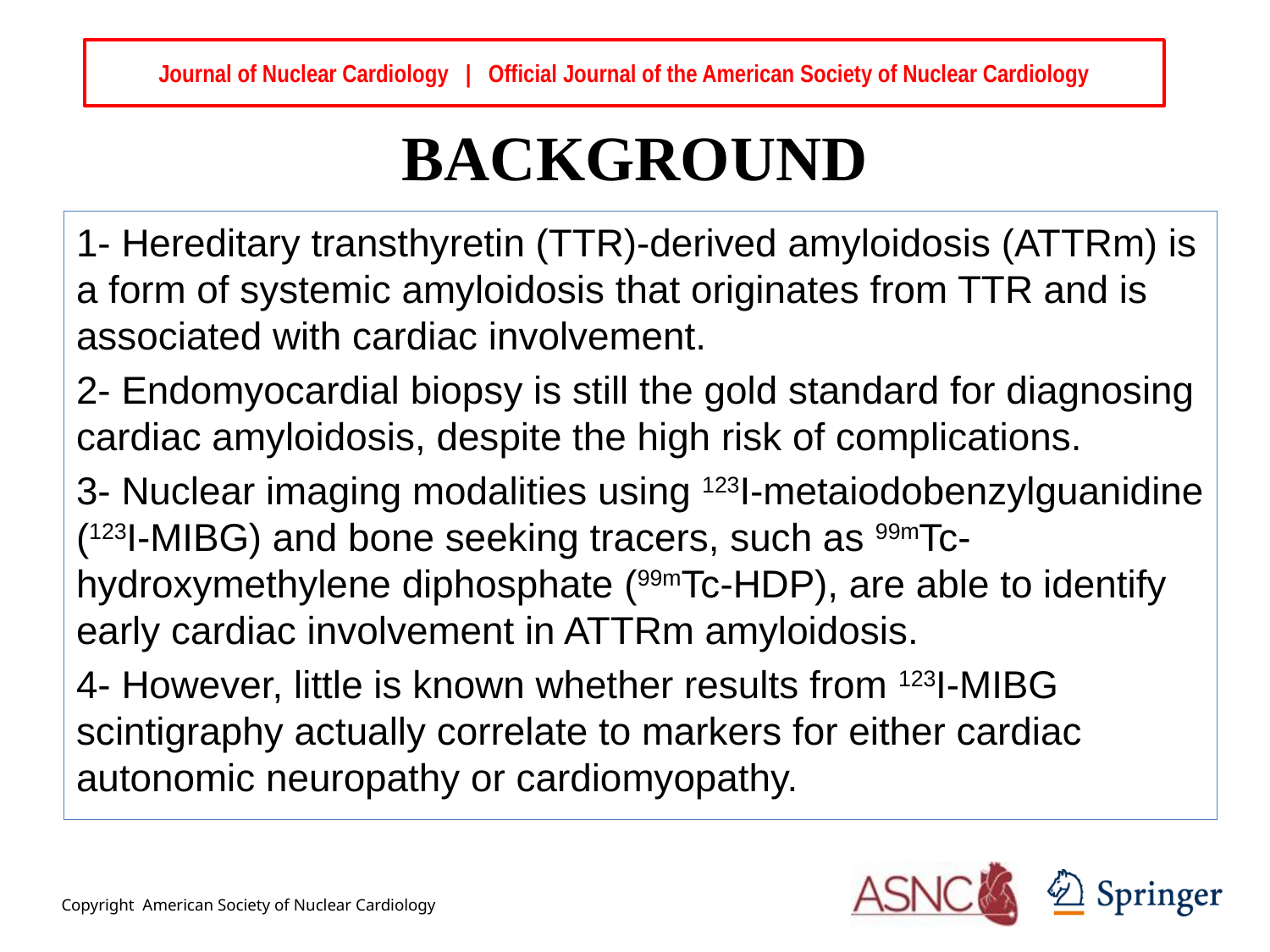

Journal of Nuclear Cardiology | Official Journal of the American Society of Nuclear Cardiology
# BACKGROUND
1- Hereditary transthyretin (TTR)-derived amyloidosis (ATTRm) is a form of systemic amyloidosis that originates from TTR and is associated with cardiac involvement.
2- Endomyocardial biopsy is still the gold standard for diagnosing cardiac amyloidosis, despite the high risk of complications.
3- Nuclear imaging modalities using 123I-metaiodobenzylguanidine (123I-MIBG) and bone seeking tracers, such as 99mTc-hydroxymethylene diphosphate (99mTc-HDP), are able to identify early cardiac involvement in ATTRm amyloidosis.
4- However, little is known whether results from 123I-MIBG scintigraphy actually correlate to markers for either cardiac autonomic neuropathy or cardiomyopathy.
Copyright American Society of Nuclear Cardiology

## Slide 3
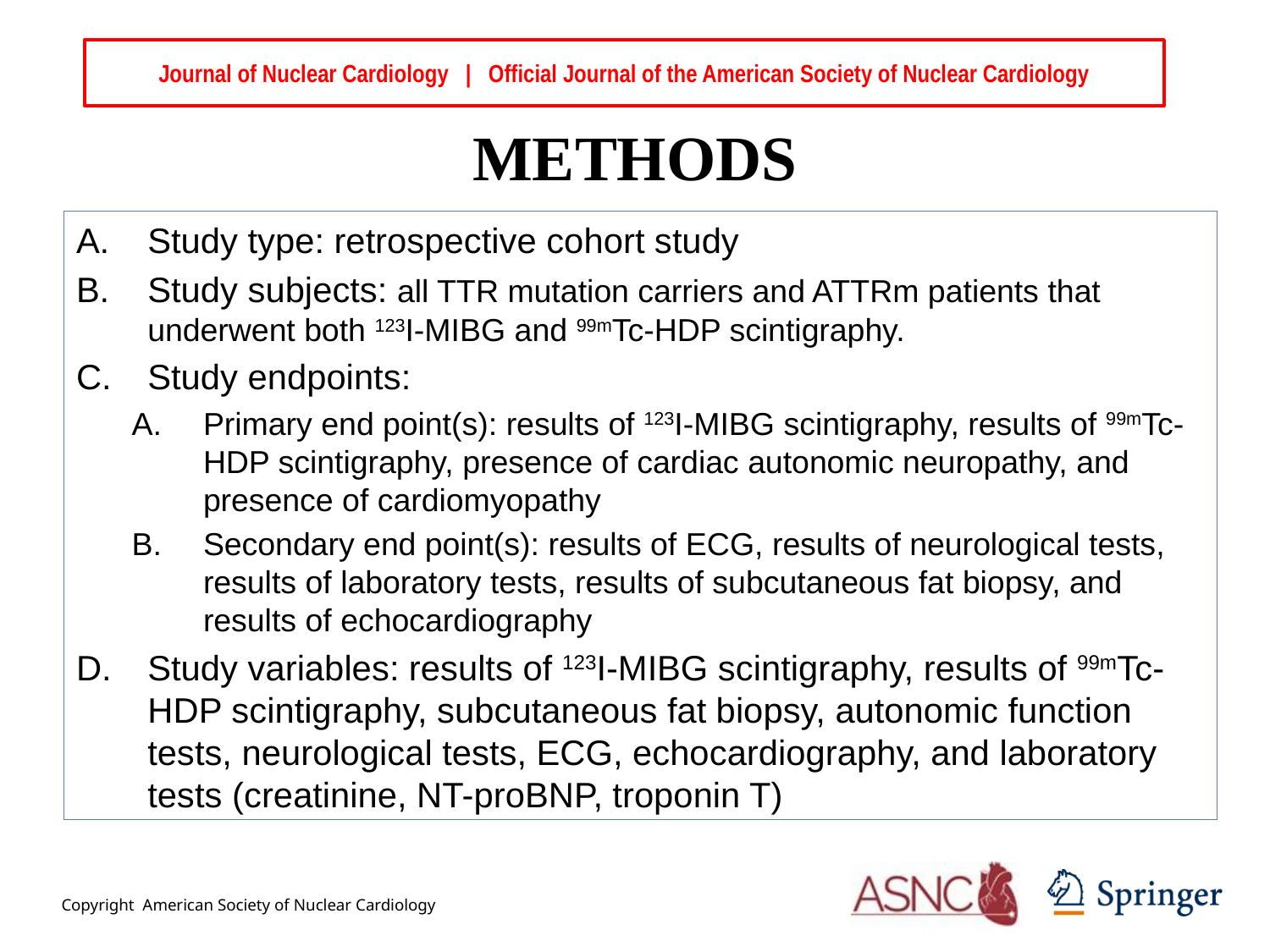

Journal of Nuclear Cardiology | Official Journal of the American Society of Nuclear Cardiology
# METHODS
Study type: retrospective cohort study
Study subjects: all TTR mutation carriers and ATTRm patients that underwent both 123I-MIBG and 99mTc-HDP scintigraphy.
Study endpoints:
Primary end point(s): results of 123I-MIBG scintigraphy, results of 99mTc-HDP scintigraphy, presence of cardiac autonomic neuropathy, and presence of cardiomyopathy
Secondary end point(s): results of ECG, results of neurological tests, results of laboratory tests, results of subcutaneous fat biopsy, and results of echocardiography
Study variables: results of 123I-MIBG scintigraphy, results of 99mTc-HDP scintigraphy, subcutaneous fat biopsy, autonomic function tests, neurological tests, ECG, echocardiography, and laboratory tests (creatinine, NT-proBNP, troponin T)
Copyright American Society of Nuclear Cardiology

## Slide 4
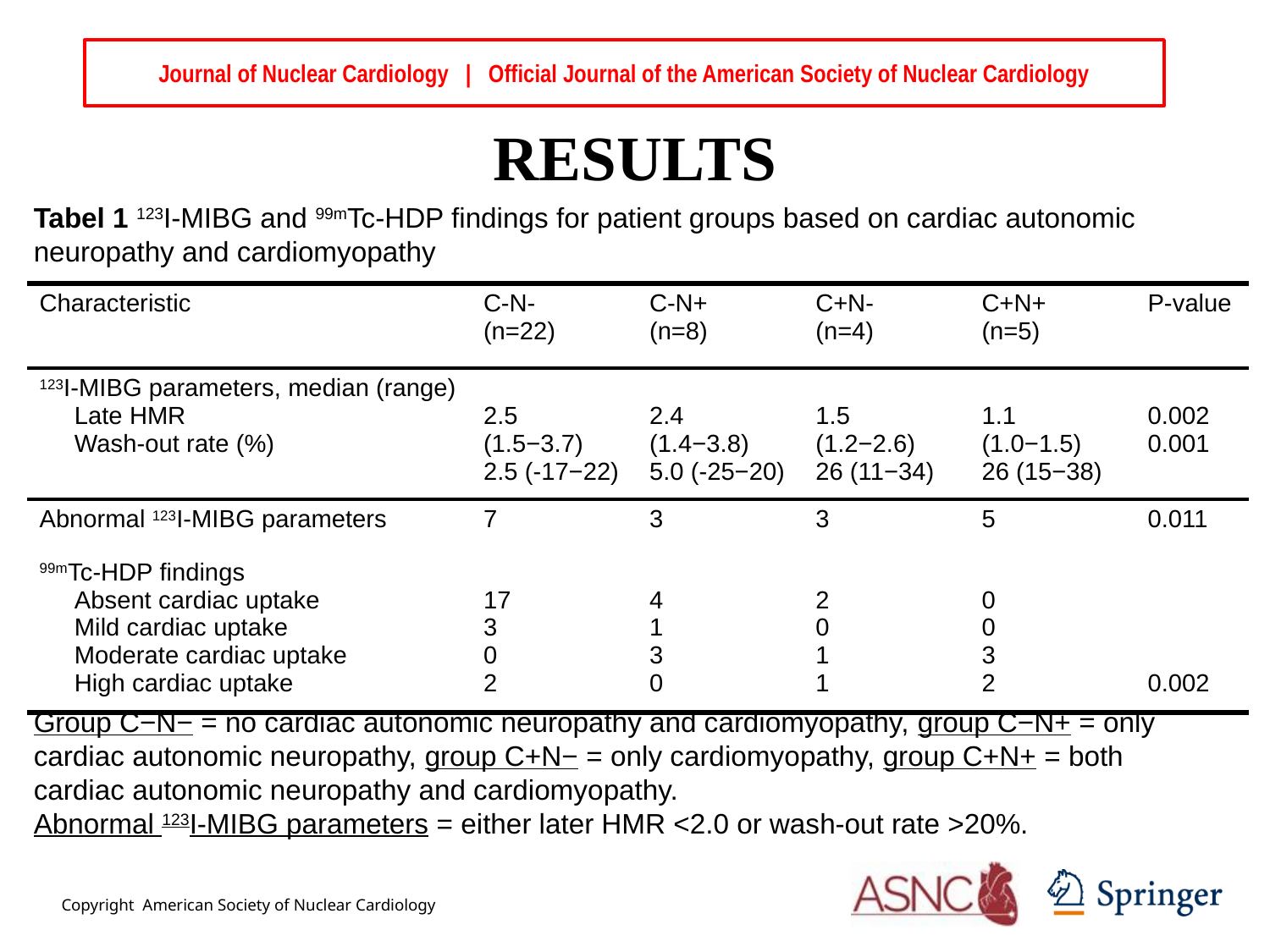

Journal of Nuclear Cardiology | Official Journal of the American Society of Nuclear Cardiology
# RESULTS
Tabel 1 123I-MIBG and 99mTc-HDP findings for patient groups based on cardiac autonomic neuropathy and cardiomyopathy
| Characteristic | C-N- (n=22) | C-N+ (n=8) | C+N- (n=4) | C+N+ (n=5) | P-value |
| --- | --- | --- | --- | --- | --- |
| 123I-MIBG parameters, median (range) Late HMR Wash-out rate (%) | 2.5 (1.5−3.7) 2.5 (-17−22) | 2.4 (1.4−3.8) 5.0 (-25−20) | 1.5 (1.2−2.6) 26 (11−34) | 1.1 (1.0−1.5) 26 (15−38) | 0.002 0.001 |
| Abnormal 123I-MIBG parameters | 7 | 3 | 3 | 5 | 0.011 |
| 99mTc-HDP findings Absent cardiac uptake Mild cardiac uptake Moderate cardiac uptake High cardiac uptake | 17 3 0 2 | 4 1 3 0 | 2 0 1 1 | 0 0 3 2 | 0.002 |
Group C−N− = no cardiac autonomic neuropathy and cardiomyopathy, group C−N+ = only cardiac autonomic neuropathy, group C+N− = only cardiomyopathy, group C+N+ = both cardiac autonomic neuropathy and cardiomyopathy.
Abnormal 123I-MIBG parameters = either later HMR <2.0 or wash-out rate >20%.
Copyright American Society of Nuclear Cardiology

## Slide 5
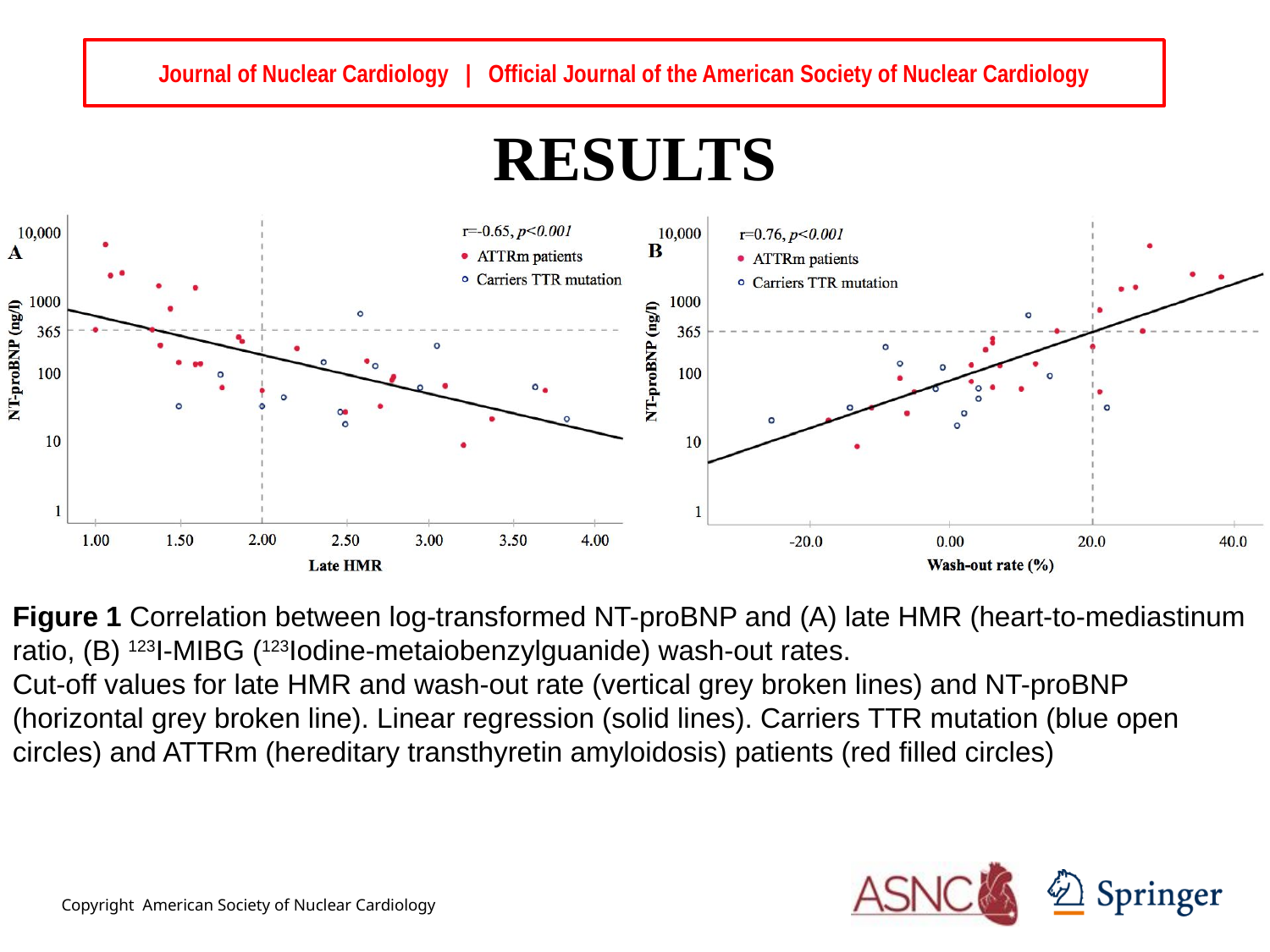

Journal of Nuclear Cardiology | Official Journal of the American Society of Nuclear Cardiology
# RESULTS
Figure 1 Correlation between log-transformed NT-proBNP and (A) late HMR (heart-to-mediastinum ratio, (B) 123I-MIBG (123Iodine-metaiobenzylguanide) wash-out rates.
Cut-off values for late HMR and wash-out rate (vertical grey broken lines) and NT-proBNP (horizontal grey broken line). Linear regression (solid lines). Carriers TTR mutation (blue open circles) and ATTRm (hereditary transthyretin amyloidosis) patients (red filled circles)
Copyright American Society of Nuclear Cardiology

## Slide 6
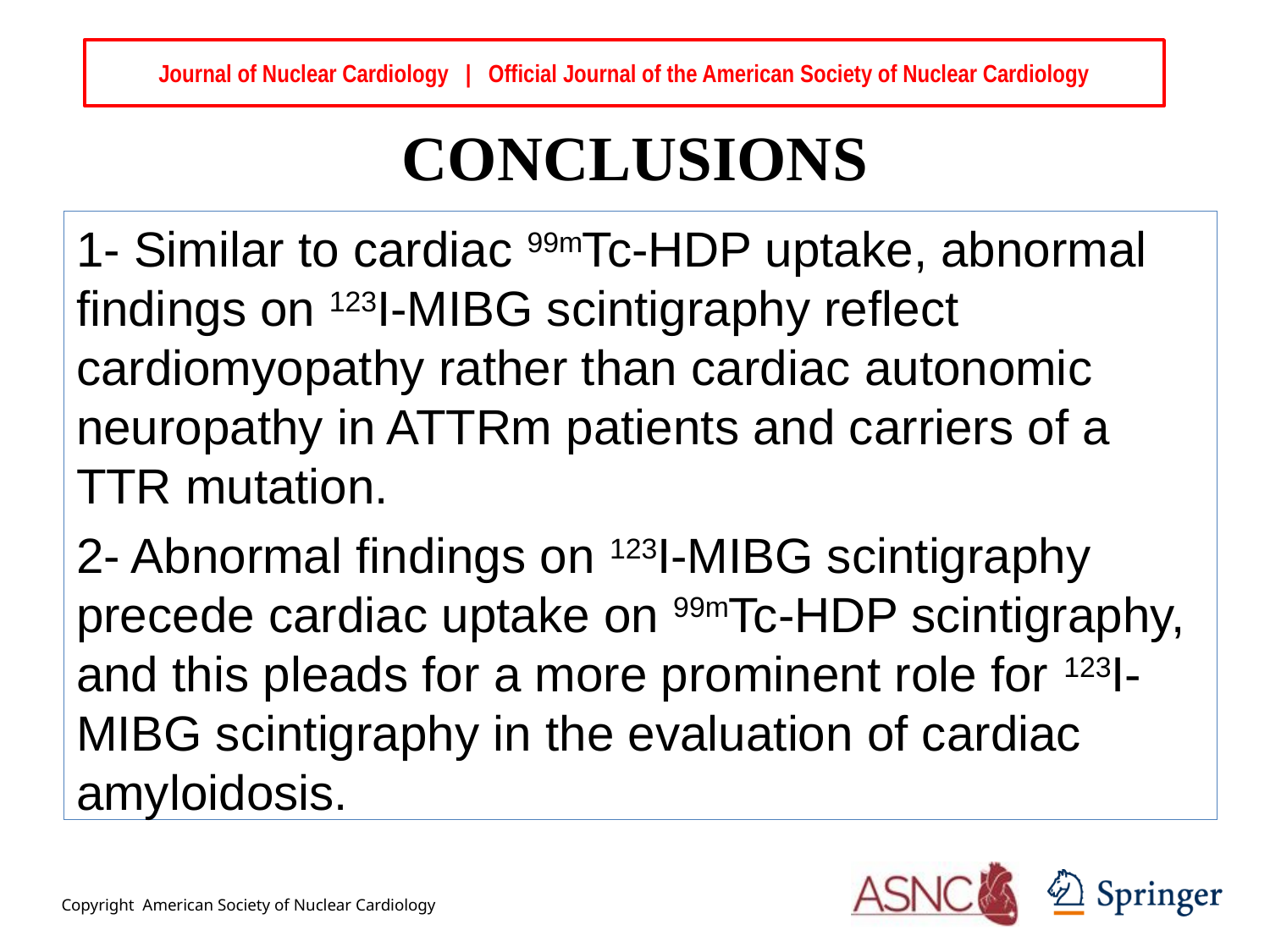

Journal of Nuclear Cardiology | Official Journal of the American Society of Nuclear Cardiology
# CONCLUSIONS
1- Similar to cardiac 99mTc-HDP uptake, abnormal findings on 123I-MIBG scintigraphy reflect cardiomyopathy rather than cardiac autonomic neuropathy in ATTRm patients and carriers of a TTR mutation.
2- Abnormal findings on 123I-MIBG scintigraphy precede cardiac uptake on 99mTc-HDP scintigraphy, and this pleads for a more prominent role for 123I-MIBG scintigraphy in the evaluation of cardiac amyloidosis.
Copyright American Society of Nuclear Cardiology
